# Supplementary material for: Brain Functional Correlates of Episodic Memory Using an Ecological Free Recall Task
Source: Brain Sci. 2021 Jul 9;11(7):911. doi: 10.3390/brainsci11070911 (PMC8303916; doi:10.3390/brainsci11070911)
Supplement: Supplementary file 1 [file brainsci-11-00911-s001.zip › brainsci-1210572-supplementary.pdf]

Brain functional correlates of episodic memory using an ecological free-recall task

Francesco Neri[1], Stefano F. Cappa[2,3], Lucia Mencarelli[1], Davide Momi[1], Emiliano Santarnecchi[4,5^], Simone Rossi[1^]

1. Siena Brain Investigation and Neuromodulation Lab (Si-BIN Lab), Unit of Neurology and Clinical Neurophysiology, Department of Medicine, Surgery and Neuroscience, University of Siena, Italy
2. Institute for Advanced Study, IUSS, Pavia, Italy
3. IRCCS Mondino Foundation, Pavia, Italy
4. Berenson-Allen Center for Noninvasive Brain Stimulation, Beth Israel Deaconess Medical Center, Harvard Medical School, Boston, MA, USA
5. Department of Cognitive Neurology, Harvard Medical School, Boston, USA

^ co-senior authors of the manuscript

1) Methods

2) Results

Table

Supplementary Table S1

Supplementary Table S2

Supplementary Table S3

Supplementary Table S4

Figure

Supplementary Figure S1

Supplementary Figure S2

Methods

Each passage was registered in Italian language. As an example, we report a passage that was listened by participants (n.1 of 8 possible prose passages). Each "concept" is shown between two vertical bars:

| Una malattia misteriosa | acceca | i daini | che vivono nei boschi |dell'Appennino Toscano. | Un virus o un veleno | non ancora identificato | provoca la distruzione del nervo ottico di questi timidi animali, | che in breve divengono incapaci di provvedere a se stessi, | precipitano nei dirupi | o vengono investiti dalle auto. | In un anno | nelle province di Pistoia | e Firenze | sono stati raccolti dieci esemplari ciechi. | Cinque nel solo mese di maggio. | Gli specialisti parlano di epidemia dilagante. | La causa della malattia è ancora sconosciuta. | I sintomi fanno pensare ad un grave avvelenamento | da metalli pesanti. | Forse questi animali hanno bevuto acqua inquinata | da discariche abusive di pile e batterie | che contengono piombo e cadmio. | La procura circondariale di Firenze ha aperto un'inchiesta | per fare luce sulle cause dell'epidemia. | Si teme che anche altri animali possano ammalarsi, | ma comunque la situazione appare abbastanza circoscritta | e sembra che non ci sia pericolo di avvelenamento | per gli abitanti della zona, | i quali comunque sono molto preoccupati. |

Total concepts: 30

| A mysterious disease | blinds | the fallow deer | living in the woods | of the Tuscan Apennines. | A virus or poison | not yet identified | causes destruction of the optic nerve of these shy animals, | who soon become unable to provide for themselves, | fall into the cliffs | or are hit by cars. | In one year | in the provinces of Pistoia | and Florence | ten blind fallow deer were collected. | Five in the month of May alone. | Specialists speak of a rampant epidemic. | The cause of the disease is still unknown. | Symptoms suggest severe poisoning | from heavy metals. | Perhaps these animals drank polluted water | from illegal dumps of cells and batteries | which contain lead and cadmium. | The district attorney of Florence has opened an investigation | to shed light on the causes of the epidemic. | There are fears that other animals may also get sick, | but the situation appears quite limited | and it seems that there is no danger of poisoning | for the inhabitants of the area, | who are still very worried. |

Total concepts: 30

Results

Table S1. Questionnaire results.

	FR/IR similarity	*FR/DR similarity*	*FR Ability*	*Thinking Interference*	*fMRI Noise Interference*	*BC Ability*	*Tasks Interference*	*Shifting Ability*	*Visual Imagination*
Mean	5,43	5,79	5,79	1,79	4,00	4,64	1,00	5,43	5,64
SD	0,76	0,97	0,58	1,19	2,04	1,78	0,00	1,28	1,01
Median	5	6	6	1	5	5	1	5,5	6
Freq. 1	0	0	0	8	2	0	14	0	0
Freq. 2	0	0	0	3	3	3	0	0	0
Freq. 3	0	0	0	2	1	1	0	2	0
Freq. 4	1	1	4	0	0	1	0	0	2
Freq. 5	7	5	9	1	3	4	0	5	4
Freq. 6	5	4	1	0	5	3	0	4	5
Freq. 7	1	4	0	0	0	0	0	3	3
Total	**14**	**14**	**14**	**14**	**14**	**14**	**14**	**14**	**14**

Table S2. MNI coordinates for each region showing increased BOLD signal in the BC>FR contrast.

**Activation Loci (BC>FR Contrast)**	**Cluester size (voxels)**	**T**	**Coordinates (MNI)**		
			**x**	**y**	**z**
Right & Left Parietal Cortex	17011				
L Inferior Parietal Lobule		18,04	-44	-40	44
R Superior Parietal Lobule		17,46	18	-70	58
R Inferior Parietal Lobule		18,46	50	-38	52
Right Frontal Cortex	3590				
R IFG (p. Triangularis)		13,15	50	12	26
R Middle Frontal Gyrus		12,04	46	42	18
R IFG (pars opercularis)		6,53	42	60	-4
Right Frontal Cortex	1472				
R Middle Frontal Gyrus		14,50	30	6	58
Left Cerebllum	1001				
L Cerebellum (Crus 1)		8,30	-8	-80	-20
L Cerebellum (Crus 1)		7,48	-30	-70	-26
Left Temporal Cortex	915				
L Inferior Temporal Gyrus		14,50	-52	-58	-16
Cingulate Cortex	700				
R Medial Cingulate Cortex		9,03	6	14	50
Right Temporal Cortex	699				
R Inferior Temporal Gyrus		14,70	58	-54	-14
Right Cerebellum	277				
R Cerebellum (Crus 1)		7,64	30	-66	-30
Right Frontal Cortex	274				
R IFG (p. Orbitalis)		8,11	34	20	-4
Right Cerebellum	199				
R Cerebellum (VIII)		10,30	18	-76	-50
Left Cerebellum	195				

L Cerebellum		11,23	-28	-74	-52
Right & Left Cerebellum	71				
Cerebellum		6,45	0	-50	-22
Right Thalamus	69				
R Thalamus		6,14	8	-18	8

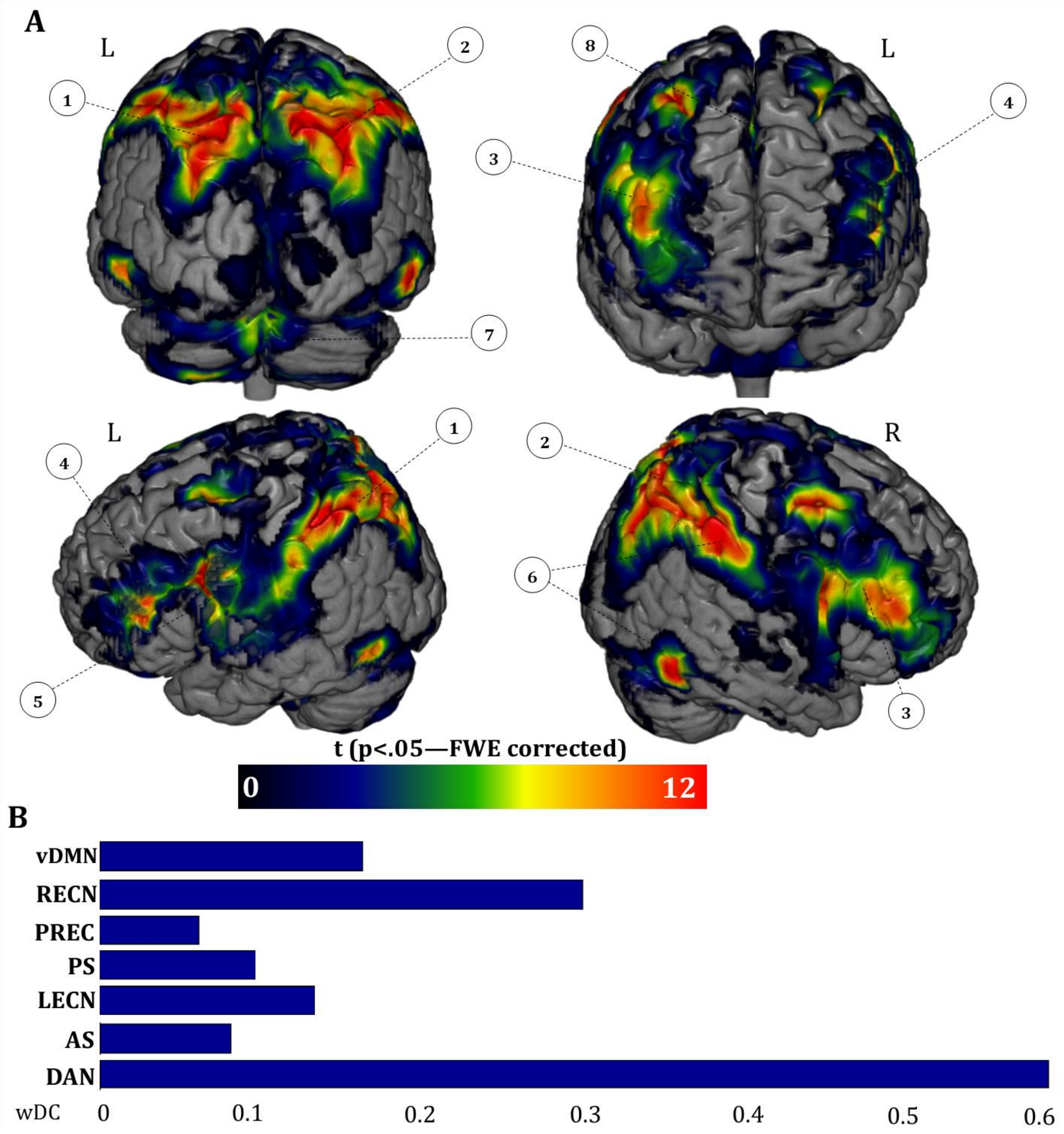

Figure S1. Threshold activation maps of the BC>FR contrast. Whole brain activation is displayed on the 3D rendered MNI reference brain (statistical threshold of p<.05 FWE-corrected; cluster size: >50) **1:** Left Parietal Cortex; **2:** Right Parietal Cortex; **3:** Right Frontal Cortex **4:** Left Frontal Cortex; **5:** Left Temporal Cortex; **6:** Right Temporal Cortex; **7**: Bilateral Cerebellum; **8**: Cingulate Cortex. (**A**). Activation overlap with the RSNs is shown (**B**).

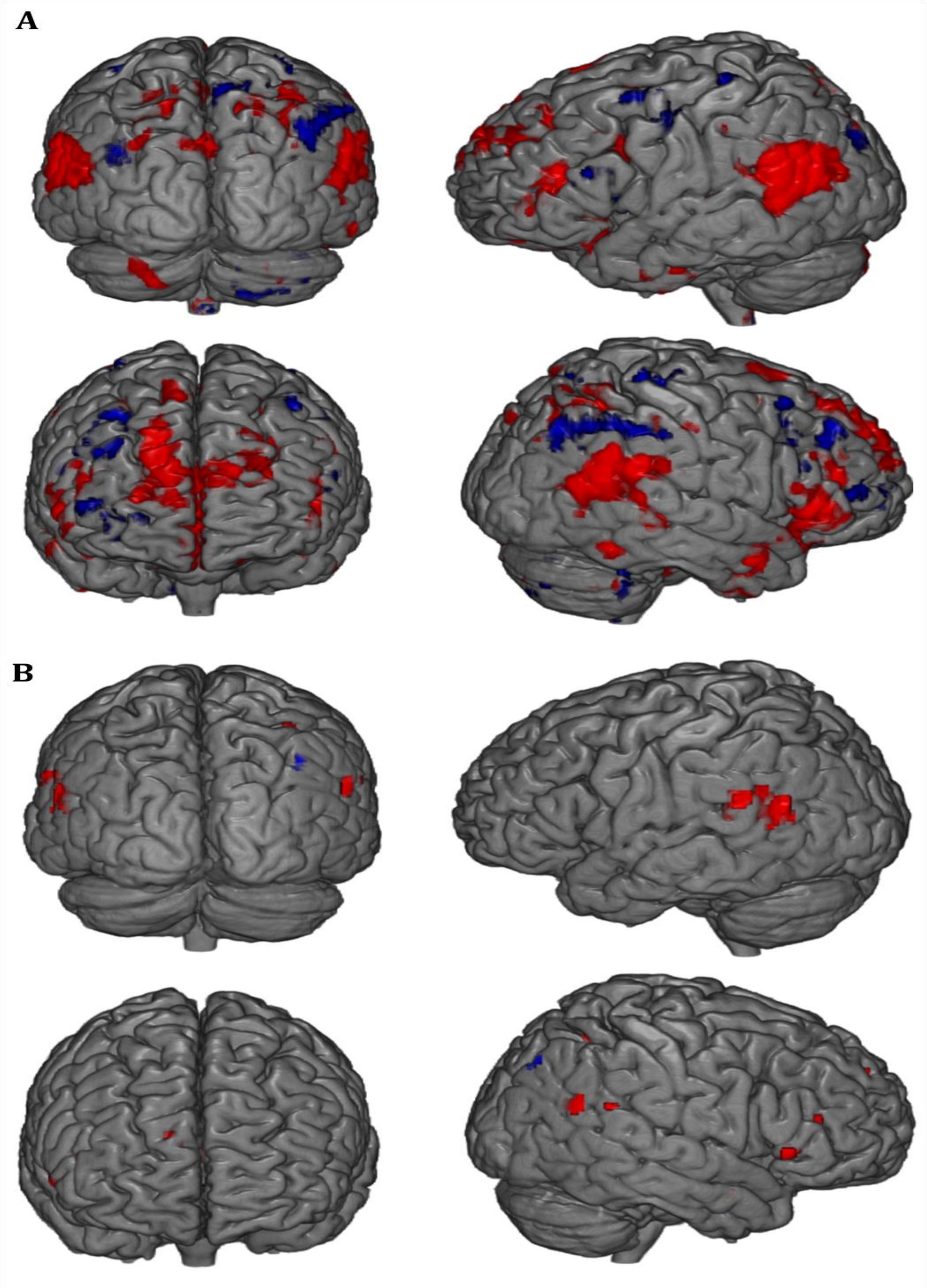

Figure S2. Threshold activation maps of the BC>FR contrast, two sided of the MVPA analysis. Height threshold = p<.05; uncorrected; cluster-size FDR corrected p<.05 **(A**). Height threshold = p<.05; FDR corrected; cluster-size FDR corrected p<.05 **(B).**

Table S3. Results Table of MVPA analysis. Height threshold = p<.05; uncorrected; cluster-size FDR corrected p<.05

X	Y	Z	size	size p-FWE	size p-FDR	size p-unc	peak p-FWE	peak p-unc
-46	-58	16	10757	0.000000	0.000000	0.000000	0.005171	0.000000
10	54	-6	8416	0.000000	0.000000	0.000000	0.023910	0.000000
56	26	2	7764	0.000000	0.000000	0.000000	0.164791	0.000001
-54	10	36	1310	0.000000	0.000000	0.000000	0.999999	0.000020
-32	-88	36	884	0.000000	0.000000	0.000000	1	0.000040
48	38	18	585	0.000023	0.000001	0.000000	1	0.000022
-46	-38	38	435	0.000568	0.000030	0.000000	1	0.000112
-22	-84	-38	347	0.004402	0.000205	0.000003	1	0.000070
16	-36	-38	321	0.008315	0.000345	0.000006	1	0.000573
32	-46	-6	285	0.020583	0.000774	0.000016	1	0.000400
60	-52	-16	274	0.027314	0.000929	0.000021	1	0.000075
36	-22	58	271	0.029520	0.000929	0.000023	1	0.000116
24	26	2	251	0.049767	0.001461	0.000039	1	0.001137
-26	10	54	208	0.155371	0.004489	0.000128	1	0.000071
-4	-48	-66	195	0.218310	0.006111	0.000187	1	0.000538
28	-76	-42	191	0.242017	0.006446	0.000210	1	0.000086
22	-82	-28	179	0.327382	0.008682	0.000301	1	0.000018
24	50	-8	176	0.352283	0.008980	0.000329	1	0.000884
38	34	32	173	0.378649	0.009321	0.000361	1	0.000029
-36	-30	66	169	0.416064	0.010011	0.000408	1	0.001867
-38	-24	0	148	0.647117	0.018461	0.000790	1	0.000938
-36	-30	66	169	0.416064	0.010011	0.000408	1	0.001867
-38	-24	0	148	0.647117	0.018461	0.000790	1	0.000938

Table S4. Results Table of MVPA analysis. Height threshold = p<.05; FDR corrected; cluster-size FDR corrected p<.05

X	Y	Z	size	size p-FWE	size p-FDR	size p-unc	peak p-FWE	peak p-unc
-46	-58	16	343	0.000000	0.000000	0.000000	0.005171	0.000000
0	-18	36	56	0.000000	0.000000	0.000000	0.147889	0.000001
60	-58	26	34	0.000000	0.000000	0.000000	0.357044	0.000001
10	54	-6	28	0.000000	0.000000	0.000000	0.023910	0.000000
56	26	2	24	0.000002	0.000000	0.000000	0.164791	0.000001
10	52	34	20	0.000011	0.000003	0.000000	0.624534	0.000003
-12	-68	34	13	0.000443	0.000093	0.000015	0.996944	0.000006
50	-40	2	11	0.001429	0.000262	0.000048	0.630946	0.000003
36	-52	56	8	0.009552	0.001406	0.000319	0.994514	0.000005
40	-78	44	8	0.009552	0.001406	0.000319	0.999962	0.000013
8	-58	38	5	0.080245	0.009424	0.002784	0.999994	0.000016
0	40	22	5	0.080245	0.009424	0.002784	1	0.000037
0	32	0	5	0.080245	0.009424	0.002784	0.999997	0.000017
-2	-48	38	4	0.171926	0.014542	0.006279	0.999988	0.000015
-4	-44	30	4	0.171926	0.014542	0.006279	0.999956	0.000012
48	38	18	4	0.171926	0.014542	0.006279	1	0.000022
-48	-30	-2	4	0.171926	0.014542	0.006279	0.999957	0.000012
56	4	-22	4	0.171926	0.014542	0.006279	1	0.000026
22	-82	-28	4	0.171926	0.014542	0.006279	0.999998	0.000018
36	-50	38	3	0.366853	0.029104	0.015213	1	0.000021
-42	-2	30	3	0.366853	0.029104	0.015213	1	0.000021
62	-44	26	3	0.366853	0.029104	0.015213	1	0.000036
52	2	-28	3	0.366853	0.029104	0.015213	1	0.000037
